# A challenging case of drug‐related acute fibrinous and organizing pneumonia: A rare case report

**DOI:** 10.1002/rcr2.1357

**Published:** 2024-04-24

**Authors:** Chenxia Guo, Wei Wu, Xiang Zhu, Qingtao Zhou, Ying Liang

**Affiliations:** ^1^ Department of Respiratory and Critical Care Medicine Peking University Third Hospital Beijing People's Republic of China; ^2^ Department of Pathology Peking University Third Hospital Beijing People's Republic of China

**Keywords:** acute fibrinous and organizing pneumonia (AFOP), apalutamide, interstitial lung disease, leuprorelin acetate, prostate cancer

## Abstract

A 65‐year‐old man presented with intermittent fever and progressive shortness of breath. He responded poorly to antibiotics and corticosteroids (methylprednisolone 40 mg/d). Chest computed tomography scans showed diffuse consolidations and ground glass density patchy opacities in both lungs and these lesions progressed rapidly. The diagnosis of acute fibrinous and organizing pneumonia (AFOP) was confirmed through transbronchial cryobiopsy. This patient had prostate cancer with bone metastasis for 4 months and took the anti‐prostate cancer medications including apalutamide and leuprorelin acetate. Considering his medication history, the patient was diagnosed with AFOP induced by anti‐prostate cancer medications through panel discussion of multidisciplinary teams. Intravenous methylprednisolone of 500 mg/day was administered for 3 days and then slowly tapered. The patient's shortness of breath gradually subsided. In addition, the lesions in the lungs improved significantly on follow up imaging. AFOP induced by anti‐prostate cancer medications is rare. To our knowledge, this is the first reported case and high‐dose glucocorticoid treatment may be required in some of these cases.

## Graphical abstract

Acute fibrinous and organizing pneumonia (AFOP) is a rare type of interstitial pneumonia. We present a case of AFOP probably induced by anti‐prostate cancer medications.

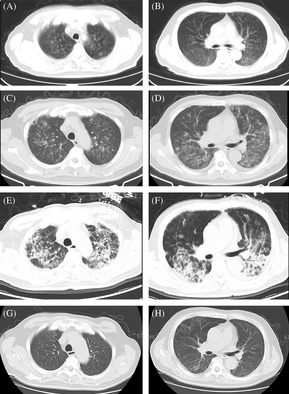

## INTRODUCTION

Acute Fibrinous and Organizing Pneumonia (AFOP) is a rare type of interstitial pneumonia that can be classified into idiopathic and secondary forms. The latter can be caused by various factors such as infections, autoimmune diseases, adverse drug reactions, occupational and environmental exposure.^1^ AFOP can affect patients of all ages, although it is slightly more common in males. Diagnosis of AFOP requires the exclusion of other acute lung injury lesions such as acute respiratory distress syndrome (ARDS), diffuse alveolar damage and eosinophilic pneumonia.^1^, ^2^ It is typically characterized by diffuse or patchy distribution of ground‐glass opacities (GGO) and/or consolidations in the lungs on chest imaging. Unlike any known interstitial pneumonia, richly fibrinous inflammatory exudate within the alveoli and in the respiratory tract, along with the formation of fibrin conglomerates and organizing pneumonia, can be seen in the alveolar cavity under the microscope, without the presence of hyaline membranes, eosinophilia, bronchiolitis, or granulomas. Diagnosis of AFOP is based on clinical features, such as fever, cough and dyspnea, as well as characteristic computed tomography (CT) imaging findings. There are no standard diagnostic criteria for AFOP at present, and the diagnosis mainly depends on characteristic pathological findings. The case reports of AFOP are limited and AFOP induced by anti‐prostate cancer drugs is rare.

## CASE REPORT

**FIGURE 1:**
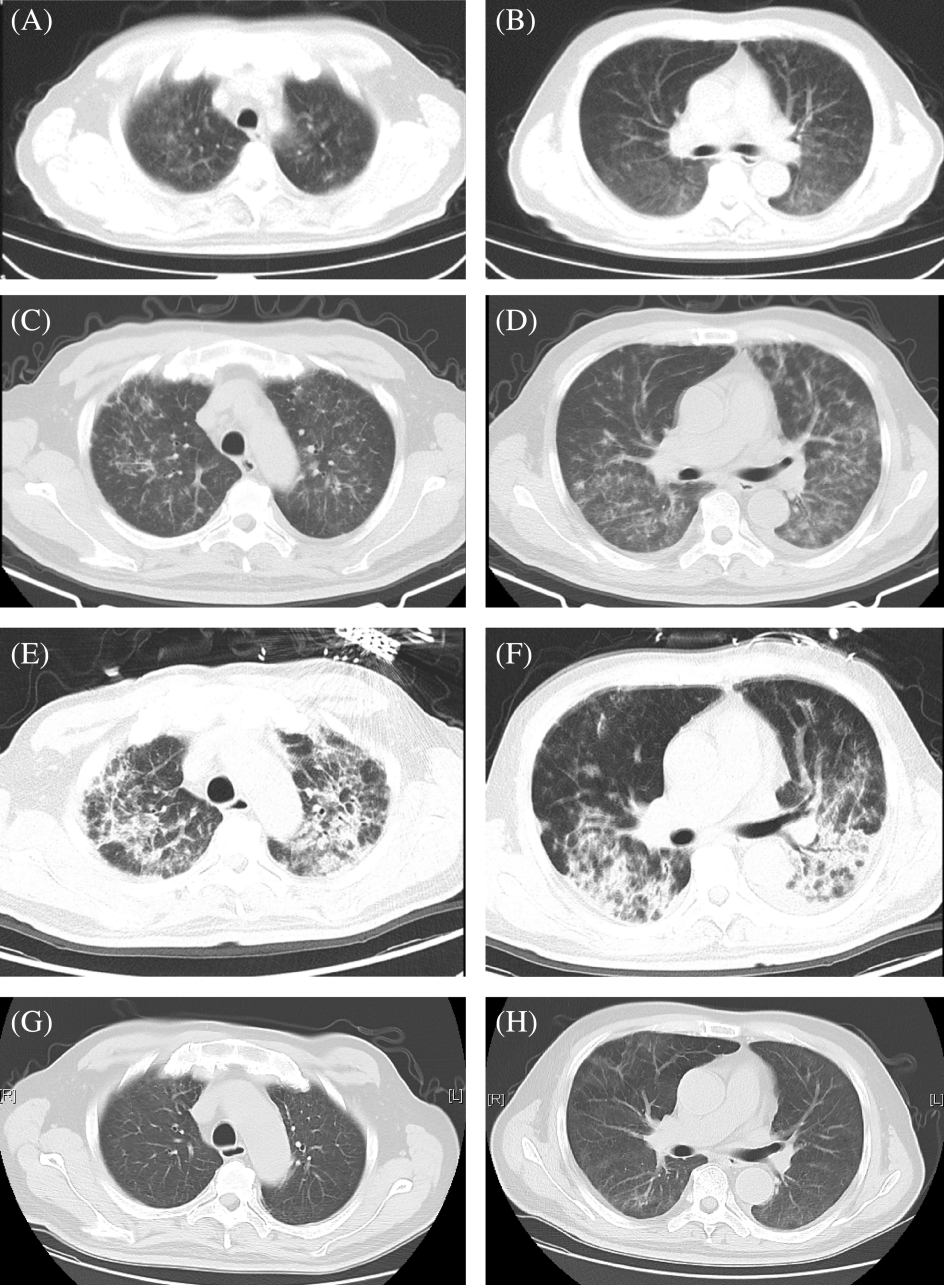
Chest computed tomography (CT) scans (A) and (B) show ground glass density patchy opacities in the lungs on April 2, 2023. Chest CT scans (C) and (D) show multiple nodules and patchy lesions are distributed along the bronchovascular bundles in both lungs and the lesions are aggravated on April 7, 2023. PET‐CT scans (E) and (F) show the lesions in the lungs are further aggravated on April 15, 2023, with more consolidation distributed in the upper and lower lungs. Chest CT scans (G) and (H) on show the lesions are significantly improved on May 31, 2023.

A 65‐year‐old man was admitted to the hospital after a 12‐day history of intermittent fever and chills, exertional dyspnea, and dry cough despite anti‐infective therapy; his symptoms were significantly worse 5 days prior to admission. His temperature remained elevated, leading to admission to our fever clinic. Blood tests showed a leukocyte count of 7.44 × 10^9^/L, with neutrophil, lymphocyte, and eosinophil percentages of 75.4%, 15.1%, and 0.2%, respectively. Chest computed tomography (CT) scans showed diffuse consolidations and ground‐glass density patchy opacities in both lungs (Figure 1A,B). Pneumonia was suspected, and the patient was treated with piperacillin sulbactam and sulphanilamide/trimethoprim, but with no improvement. The patient's dyspnea worsened, with a decrease in the PaO_2_/FiO_2_ ratio and progression of lesions on chest CT scans (Figure 1C,D). He was then admitted to our respiratory intensive care unit for further treatment.

He had prostate cancer with bone metastasis for 4 months and had been treated with four apalutamide capsules daily for 2 months. Additionally, he had received a monthly sustained‐release injection of leuprorelin acetate microspheres subcutaneously at 3.75 mg from December 2022 to February 2023 and at 11.25 mg in March 2023.

On physical examination, his temperature was 37.4°C, pulse 78 bpm, respiratory rate 18 bpm, blood pressure 106/73 mmHg. No swollen superficial lymph nodes were detected in the neck, clavicular and groin areas. Respiration sounds were clear in the lungs, and wheezing sound and crackles were not heard. His SpO_2_ on room air was 88%.

**FIGURE 2:**
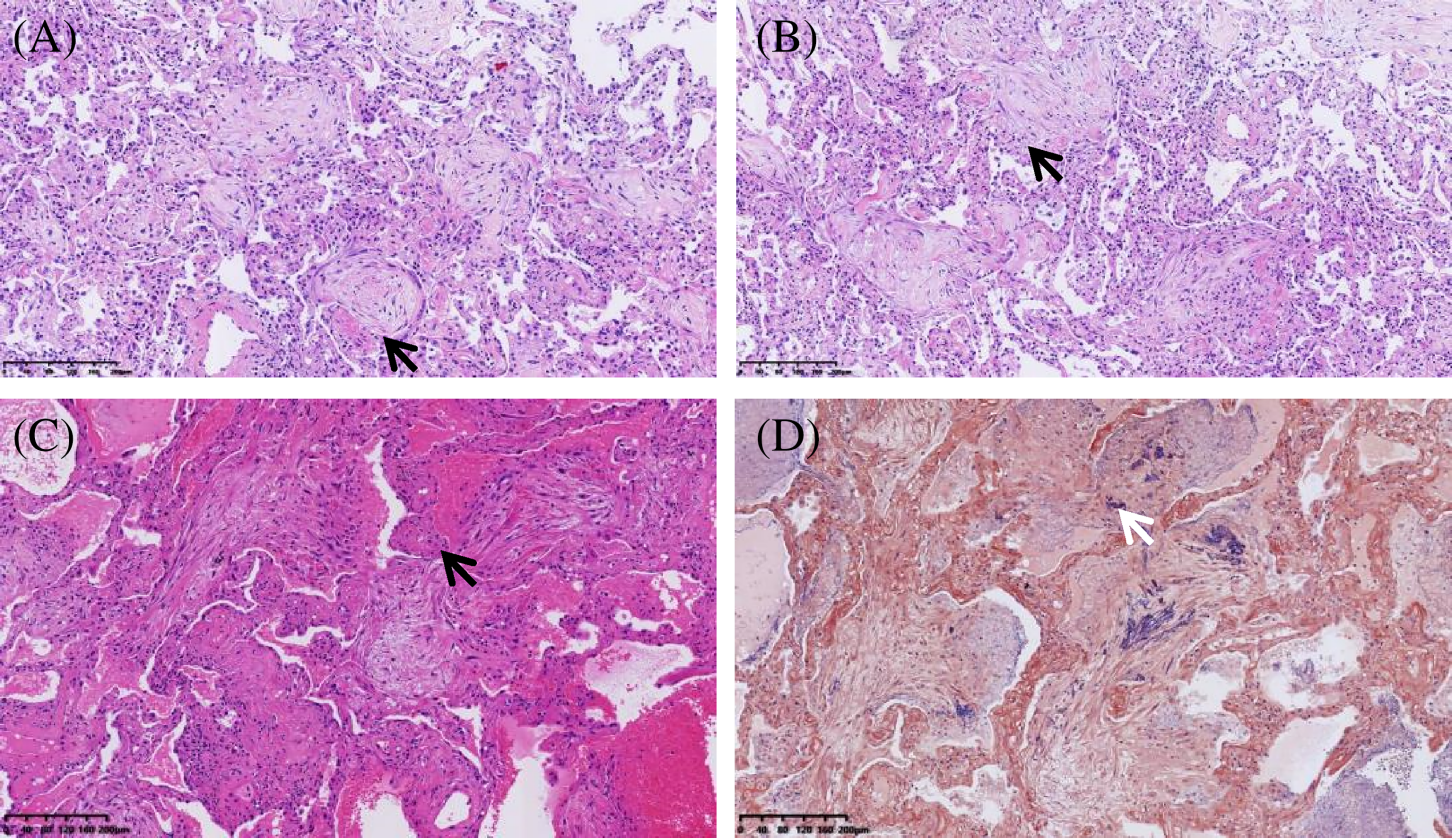
(A‐C) Histological findings obtained by TBCB show organizing pneumonia and foci of fibrin (fibrin balls) within the Masson bodies (black arrow) in the alveoli, which is consistent with the characteristics of AFOP. (D) PTAH (phosphotungstic acid‐haematoxylin) staining shows intra‐alveolar blue fibrin deposition (white arrow).

Upon admission on April 8, 2023, the patient was treated with supplemental oxygen therapy via a nasal cannula at a rate of 6 L/min, intravenous infusion of methylprednisolone (40 mg/d), and empirical antibiotic treatment. Various tests were performed, including procalcitonin, blood culture, biochemistry, coagulation function, urine and stool examination, thyroid function, tumour marker, autoimmune antibodies, and 1,3 β‐D‐Glucan and galactomannan. All of the tests were negative, and no clinical abnormalities were detected during metagenomic next‐generation sequencing (mNGS) detection in blood. Echocardiography and abdominal ultrasound also showed no abnormalities. Further testing was conducted through bronchoscopy and alveolar lavage in the left upper lobe of the lung. The lymphocyte percentage in bronchoalveolar lavage fluid (BALF) was 83%, and no pathogens were found in mNGS detection. Since there was no evidence of infection, antibiotics and methylprednisolone were discontinued. However, the patient still experienced dyspnea and persistent fever. A positron emission tomography (PET)‐CT examination revealed that the lesions in lungs had progressed and appeared to be inflammatory (Figure 1E,F). The results also showed prostate cancer and bone metastasis. A transbronchial cryobiopsy (TBCB) was performed in the right lower lobe, and the histopathology showed that Masson bodies were seen in some alveolar spaces, and interalveolar fibrin deposition mixed with Masson bodies were also seen in other alveolar spaces. The alveolar septum was widened in varying degrees, and interstitial infiltration of lymphocytes, monocytes and neutrophils were present (Figure 2). After a panel discussion of multidiscipline teams including respiratory medicine, urology, radiology, pathology and pharmacology, the clinical and pathological findings were consistent with acute fibrinous and organizing pneumonia (AFOP), which was probably induced by his anti‐prostate cancer drugs (apalutamide and leuprorelin). Both drugs were stopped, and glucocorticoid was given again. Methylprednisolone was started on a dose of 80 mg/day, but the dose was increased to 500 mg/day for 3 days due to worsening dyspnea and decreased PaO_2_/FiO_2_ (approximately 150 mmHg). The dose was then gradually tapered over a duration of 3.5 months until discontinuation. All his symptoms gradually improved, and was associated with significant radiologic improvement on repeat CT chest imaging. (Figure 1G,H).

## DISCUSSION

We confirmed the histological diagnosis of AFOP in this case, probably induced by anti‐prostate cancer medications. However, as a rare interstitial pneumonia, the aetiology and pathogenesis of AFOP remain complex, and there are no clear treatment guidelines available for it. In this case, we considered the possibility that AFOP was linked to endocrine therapy for prostate cancer, as anti‐prostate cancer drugs have been reported to be associated with interstitial lung disease (ILD). According to the literatures, the incidences of interstitial lung disease caused by leuprorelin and apalutamide were 0.51% and 1.14%,^3^ respectively. Androgen receptor antagonists may increase the risk of ILD.^4^, ^5^ Despite investigating the potential exposure history and other possible causes of interstitial pneumonia, we were still unable to determine the exact cause of AFOP in this case.

Additionally, there have been two severe cases of ILD caused by apalutamide treatment, which were similar to that of our patient, with progressive dyspnea after using apalutamide, rapid imaging progression, rapid decline in oxygenation, and lymphocyte dominance in BALF. Both of them were treated with initial methylprednisolone doses of 500 mg/day and showed improvement.^6^ However, the histological findings in these two cases were not AFOP. In our case, apalutamide caused more severe interstitial lung disease and required a large amount of glucocorticoid. Apalutamide has been associated with a higher incidence of ILD compared to other drugs. Therefore, we speculated that AFOP in our patient was more associated with apalutamide. To our knowledge, this was the first case of AFOP caused by anti‐prostate cancer drugs. Clinicians should be aware of various types of ILD in patients with prostate cancer who are undergoing endocrine therapy. If relevant respiratory symptoms occur, after excluding environment exposure history and other etiologies causing interstitial pneumonia, the possibility of adverse drug reactions should be considered in these patients.

## AUTHOR CONTRIBUTIONS

All authors contributed equally to this article.

## ETHICS STATEMENT

The authors declare that appropriate written informed consent was obtained for the publication of this manuscript and accompanying images.

## Data Availability

Data sharing not applicable to this article as no datasets were generated or analysed during the current study.
